# A Damage Detection Approach for Axially Loaded Beam-like Structures Based on Gaussian Mixture Model

**DOI:** 10.3390/s22218336

**Published:** 2022-10-30

**Authors:** Francescantonio Lucà, Stefano Manzoni, Francesco Cerutti, Alfredo Cigada

**Affiliations:** Department of Mechanical Engineering, Politecnico di Milano, Via La Masa, 1-20156 Milan, Italy

**Keywords:** structural health monitoring, tie-rods, beam-like structures, unsupervised learning, unsupervised data clustering, gaussian mixture model, mahalanobis squared distance, real damage

## Abstract

Axially loaded beam-like structures represent a challenging case study for unsupervised learning vibration-based damage detection. Under real environmental and operational conditions, changes in axial load cause changes in the characteristics of the dynamic response that are significantly greater than those due to damage at an early stage. In previous works, the authors proposed the adoption of a multivariate damage feature composed of eigenfrequencies of multiple vibration modes. Successful results were obtained by framing the problem of damage detection as that of unsupervised outlier detection, adopting the well-known Mahalanobis squared distance (MSD) to define an effective damage index. Starting from these promising results, a novel approach based on unsupervised learning data clustering is proposed in this work, which increases the sensitivity to damage and significantly reduces the uncertainty associated with the results, allowing for earlier damage detection. The novel approach, which is based on Gaussian mixture model, is compared with the benchmark one based on the MSD, under the effects of an uncontrolled environment and, most importantly, in the presence of real damage due to corrosion.

## 1. Introduction

Structural Health Monitoring (SHM) is certainly one of the most-discussed topics in the literature of mechanical, civil and aerospace engineering, due to the central relevance of safety, maintenance and quality. By adopting the most general definition, damage is the loss of a structure to perform its intended functions [1,2] due to unwanted changes of the geometric and material properties, boundary conditions and system connectivity with respect to an initial reference condition. An early detection of these changes plays a crucial role to carry out prompt maintenance actions [3]. For this reason, structures are monitored over time by adopting sensors to measure physical quantities that are related to the structural properties [4].

According to a data-driven approach, data coming from sensors are used to extract damage-sensitive features that can be statistically analysed to determine the current state of the system health. In many real applications, damage conditions and locations greatly vary from structure to structure and databases containing monitoring data related to all the possible damage conditions are not available. As a result, damage detection must be carried out by adopting an unsupervised learning approach (i.e., an approach that does not require damage-related data). First, monitoring data are acquired during a reference period, to statistically characterize the system response under healthy condition; damage is then assessed when a meaningful deviation from what is considered the normal behaviour is observed [2]. In real applications, unsupervised learning approaches are limited by the effects of environmental and operational variations that cause changes in the structural behaviour that, in turn, reflect in changes of the damage feature. Often, these changes are greater than those caused by damage at an early stage and they must be properly accounted for [5].

In this context, this paper is devoted to the development of unsupervised learning damage-detection approaches for beam-like structures. Beams are widely adopted in engineering structures, often representing fundamental elements of complex systems. Examples can be tie-rods of arches and vaults, diagonal braces of truss girders, struts and ties of space truss structures. More specifically, the main attention is here focused on axially loaded beams, i.e., tensioned beams. A vibration-based damage detection approach is used in this work, particularly suitable for slender structures which undergo significant vibration levels under operational conditions. Vibration-based damage detection relies on a basic assumption: the presence of damage alters the structural properties of a system (mass, stiffness, damping), which in turn, changes the dynamic response of the system [6]. Axially loaded beam-like structures can serve as a challenging case study: indeed, during their normal operation, these structures undergo dramatic changes of the axial load that reflect into significant changes of the dynamic response, thus making vibration-based damage detection a hard task.

A specific case study will be presented, which is the case of tie-rods (nevertheless, the proposed damage detection strategy can be adopted on any beam-like structure subject to axial load). Tie-rods are metallic beam elements adopted to balance the lateral force transmitted to the base of arches and vaults, both in modern and historical civil structures. The integrity of these elements is important for the overall structural equilibrium, since a tie-rod failure may result in a global failure. To fulfil their function, tie-rods are subject to a tension at the time of the installation. During their life, temperature variations cause changes in the thermal coefficients of both the tie-rod and the structure, which reflects into changes of the axial load. Other tension variations are due to deformation and displacement of the connecting walls, that may be caused by terrain crawl, subsidence of foundations or seismic events [7].

Many authors focused their attention on the axial load estimation, considering only two possible failure types: a low tension, that points out a loosen tie-rod, not exerting its intended function and therefore requiring replacement, or a high tension, pointing at a risk of overloading, due to an abnormal structural deformation. In this latter case, the tie-rod axial load can provide information about changes in the overall structural health.

When operating tie-rods are considered, there is a high uncertainty related to the real axial-load, which can be directly measured only by strain gauges or load cells, installed and calibrated prior to the initial tensioning procedure. This, of course, is not a viable solution for already installed tie-rods. For this reason, many researchers focused on indirect approaches for the identification of the tensile force [8], based on static [9,10], static–dynamic [11] or fully dynamic [12,13] in situ indirect measurements. While the main shortcomings of static and static–dynamic approaches are related to the complicated experimental procedures, fully dynamic methods are based on the estimation of the tie-rod modal parameters, which can be more easily estimated due to the slenderness of these structural elements that allows, for instance, the adoption of output-only operational modal analysis [14]. However, the problem is complex because modal parameters are not only related to the axial load but also to a number of other physical variables which are affected by a high uncertainty, such as geometrical and material properties and constraint characteristics. The inverse problem is non-linear and potentially ill-conditioned [15]; thus, many approaches that can be found in the literature are based on analytical solutions [16,17], minimization between the theoretical and the measured modal parameters [18,19], model updating [20] and genetic algorithm optimization [21].

From a review of the state of the art, all the above-mentioned references consider the excessive load or deformation as the only critical problem, neglecting any possible presence of damage in the tie-rod. On the other hand, experience shows that cracks due to ageing or corrosion phenomena can lead to high-risk situations. Often, these types of damage are hard to be detected through visual inspections, which are even made more complex by the difficulties to access tie-rods and carry out in situ tests. Moreover, as already mentioned, a change of the axial load cannot be directly related to the presence of a crack in the tie-rod, since the axial load exhibits a high sensitivity to other physical variables which are not correlated to the state of health of the tie-rod (e.g., temperature).

Recently, a strategy for damage identification in tie-rods was presented in [7], where an analysis of the tie-rod dynamic behaviour is shown and the estimate of the flexural compliance is proposed as a way to highlight the differences between a healthy and a damaged tie-rod. The potential of the approach was proved with a laboratory test where the effect of a breathing crack was reproduced by a bolted joint between two beam sections. The approach can highlight the presence of a crack but it requires a comparison with a reference tie-rod under known health conditions. Moreover, environmental and operational effects were not considered, thus the validity of the approach in field applications was not verified.

To overcome the current limitations, in previous works [22,23], the authors proposed the adoption of a vibration-based data-driven approach that does not require knowledge of any physical variable of the problem, that does not require specific in situ tests and that can be adopted continuously, without human supervision. The statistical pattern recognition approach was adopted, framing the problem of tie-rod damage detection as a multivariate outlier detection one, by adopting damage feature vectors based on modal parameters. The motivations behind the potential of the strategy are described in [22]: the most important point is that the effects of damage can be distinguished from those of the environmental variations, because they cause different changes in the pattern of modal parameters of multiple vibration modes. Consequently, a damage index was defined using a well-known multivariate metrics, the Mahalanobis squared distance (MSD). The proposed damage index showed the potential to be used for automatic outlier detection.

Starting from these observations, in [23] the authors further developed the process, focusing on the automatisation of the algorithm. A multivariate damage feature based on a collection of eigenfrequencies associated with multiple vibration modes was adopted and an automatic data cleansing algorithm was proposed, to develop a robust algorithm that can be used in real applications. The proposed strategy was validated on long term monitoring data, coming from a one-of-a-kind test case in the research field of SHM: nominally identical full-scale tie-rods, monitored for several months under the effects of uncontrolled operational and environmental conditions. Moreover, while the majority of works found in the literature consider simulated damage scenarios, a peculiarity of this work lies in the validation on data acquired during an ongoing real damage, carefully generated through a corrosion process, which realistically evolved throughout different months. The results were promising and allowed for the automatic detection of damage coming from two corrosion attacks in two different sections of the tie-rod.

This work proposes a different approach that is aimed at accounting for the dispersion associated with the data acquired under real operating conditions, through the adoption of Gaussian Mixture Model (GMM). GMM is a class of finite mixture models widely used for unsupervised data clustering, successfully adopted for applications related to sound/speech recognition [24,25] and image processing [26,27]. In the context of SHM, GMMs have been adopted for crack detection in reinforced concrete [28,29,30] and laminated composites [31] based on Acoustic Emission technique. Furthermore, GMMs have been adopted to automatically cluster features extracted from Lamb wave signals in both the time and frequency domains for aircraft wing spar damage detection under time-varying boundary condition [32]. An application to bridge monitoring is that of [33], where a GMM-based algorithm for stay cables condition monitoring is presented. The authors of [33] used GMM on a damage feature which is the ratio between the tension of multiple cables, measured through load-cells. The authors of [34] proposed the adoption of GMM based on coefficients of an autoregressive model with moving average used to fit simulated vibration data of the ASCE Benchmark Structure [35]. In [36], GMM were adopted on eigenfrequencies of the Z-24 Bridge in Switzerland, observing that the approach can outperform other state-of-the-art algorithms (principal component analysis or Mahalanobis squared distance) in the presence of non-linear effects caused by the operational and environmental conditions.

As mentioned, this paper presents a novel GMM-based damage index for SHM of axially loaded beam-like structures. To summarize, the main challenges are related to the development of a damage detection strategy that:

- Does not require knowledge of physical variables (above all, does not require axial load estimate);
- Requires a simple and cost effective set-up;
- Is automatic;
- Is validated under real environmental and operational conditions;
- Is validated in the presence of real damage.

The innovative aspects of this research are:

- The unsupervised data clustering approach to damage detection is applied for the first time to the case study of tie-rods;
- A comparison between GMM-based and MSD-based approaches is shown in the presence of a real damage condition which evolves over time. It will be shown that the proposed GMM-based approach outperforms the benchmark one, based on the classical MSD, both in terms of sensitivity and uncertainty associated with the results.

The paper is organized as it follows. In Section 2, the theoretical background and the experimental set-up are described. The damage feature based on tie-rod eigenfrequencies and the benchmark damage index are recalled in Section 2.2 and Section 2.3.1, respectively. The theoretical background on GMM and the novel damage index are described in Section 2.3.2. Results are presented in Section 3 and discussed in Section 4. The general conclusions are drawn in Section 5.

## 2. Materials and Methods

### 2.1. The Experimental Case Study

**Figure 1:**
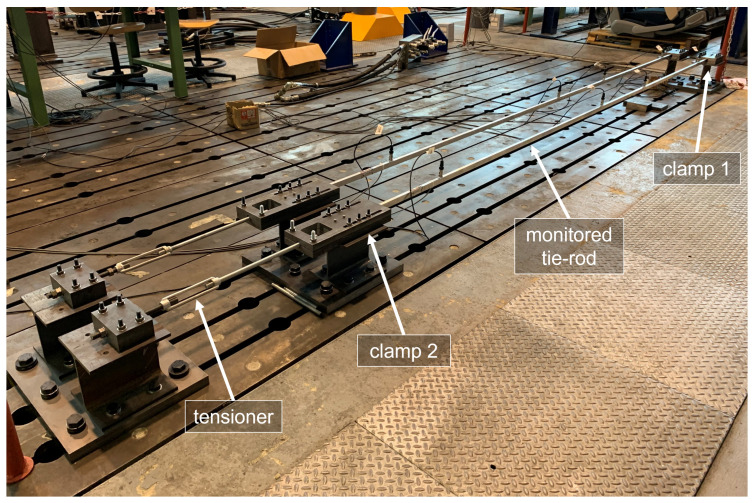
The experimental set-up in the laboratories of Politecnico di Milano.

In this section, the case study is introduced. The data that will be discussed in the following come from an experimental set-up located in the Mechanical Engineering laboratory at Politecnico di Milano, in Italy. The set-up (see Figure 1) is composed by a series of nominally identical tie-rods, monitored for long time by an SHM system, for research purposes (e.g., [22,23,37,38]).

The tie-rods are made of aluminium and they are characterized by a free length *W* of 4 m, and a cross-section of 0.015 mm × 0.025 mm. At the two ends of the beam, there are clamps made from two steel plates, in contact with the upper and lower face of the beam, held together by bolts. During the installation, the bolted joints of one of the two clamps (clamp 1 in Figure 1) are tightened, while those of the other clamp (clamp 2 in Figure 1) are left loose: in this way, the beam is not fully constrained along the axial direction, allowing traction to be applied. After the axial load is applied through a tensioner, also the bolted joints of clamp 2 are tightened, obtaining a “clamped–clamped” configuration.

The beams are equipped with general purpose industrial accelerometers, model PCB603C01, characterized by a sensitivity of 10.2 mV/(m/s${}^{2}$) and a full scale of ±490 m/s${}^{2}$. Vibration data, laboratory temperature and the axial load (which is measured through a calibrated Wheatstone full bridge) are acquired with NI9234 modules, with anti-aliasing filter on board, at a sampling frequency of 512 Hz. The resulting bandwidth of 200 Hz is appropriate to acquire the range of frequency significantly excited by the operational environment.

Indeed, the tie-rods are subject to environmental vibrations, in a laboratory where human activities take place and where other testing benches and machineries are working. The operating environment usually provides a broadband excitation that significantly decreases above 200 Hz. The temperature is not controlled: as an example, during the first year of monitoring, the temperature ranged from a minimum of 6 °C during winter, to a maximum of 29 °C during summer, with minimum and maximum daily thermal excursions approximately equal to 3 and 8 °C, respectively. The operating loads and the temperature conditions were intentionally uncontrolled, to obtain a challenging environment to test the developed unsupervised learning damage detection algorithms.

On some of the specimen, a chemical attack was carried out to intentionally introduce a corrosion process which progressively evolved through several months. A general corrosion caused a decrease of the nominal section of the tie-rod, due to a progressive electrochemical reaction between the metal and concentrated solutions of sodium hydroxide and hydrofluoric acids (more details on the corrosion process can be found in reference [23]). The data acquired during the deteriorative process will be used in Section 3, to compare the damage detection indexes.

As it will be explained in Section 2.2, this work focuses on a vibration based damage feature based on tie-rod eigenfrequencies. Before going into detail of the theory, some aspects are discussed about the eigenfrequency database that will be used in the following. Different operational modal analysis algorithms can be adopted to estimate the tie-rod modal parameters. Since the proposed approach is based on eigenfrequencies only, the choice was for the adoption of a simple single-output approach. This choice was made to develop a damage detection strategy that requires a very simple implementation and that could be easily translated to real applications. Eigenfrequencies were identified from the power spectrum of the response, adopting a single-degree-of-freedom (SDOF) modal identification technique [39], around every resonance of the tie-rod. The best fitting between the experimental power spectrum of the response, and the analytical power spectrum of the response of an SDOF system excited by white noise was carried out (see Appendix A). For this specific case, the tie-rods showed lightly coupled modes, not closely spaced in frequency and not heavily damped; thus, such simple and fast approach is a viable solution. However, it must be pointed out that the theory and approach developed in the following could be adopted regardless the operational modal analysis algorithm chosen.

The identification was carried out using vibration data acquired by a sensor that was placed at a distance approximately equal to 1/10 of the free length. This position was chosen because it is not a vibration node for the first six bending vibration modes. Despite being close to the constraints, where the eigenvector components are generally low, the signal-to-noise ratio allowed a sufficiently stable identification of the eigenfrequencies. The frequency averaging approach [40,41] was adopted to calculate the experimental power spectrum every hour, with a duration of the sub-records for the averaging procedure equal to 40 s (with an overlap of 50%), and adopting a Hanning window.

Being in an uncontrolled environment, it is not always that the averaging procedure allows obtaining a good reconstruction of the power spectrum, mainly due to the presence of disturbances. In such cases, the best fitting procedure may fail or converge to wrong solutions. For this reason, an automatic data cleansing approach was used to automatically discard the corrupted eigenfrequency estimates. The data cleansing algorithm is presented and discussed in detail in Ref. [23]; however, since the data adopted in the following are those obtained after the removal of wrong identifications, some details are provided also here, for the sake of completeness.

The data cleansing algorithm is performed at two stages, where two checks are carried out. The first check is carried out every time an eigenfrequency is identified, considering vibration modes one at a time. Outliers are discarded when a poor fit is observed between the analytical power spectrum of the response of a single-degree-of-freedom mechanical system excited by white noise [39] and the experimental power spectrum, estimated through Welch’s approach [40,41]. This can be performed by setting an acceptance threshold on the value of the $R^{2}$ coefficient (identifications associated with an $R^{2}<0.9$ were discarded in this case).

At the second stage, multiple observations of the eigenfrequencies related to all the considered vibration modes are considered, assuming that damage-related phenomena are characterized by a long-term evolution, i.e., they do not significantly evolve in a short period, e.g., one or two weeks. From analytical formulations [42,43], every tie-rod squared eigenfrequency is linearly dependent on the axial load and, consequently, squared eigenfrequencies of vibration modes are linearly dependent to each other, if the axial load is the only changing variable. This linear dependency between couples of squared eigenfrequencies is exploited, removing observations that significantly deviate from the mentioned linear trend.

### 2.2. Vibration-Based Damage Feature for Beam-Like Structures

In the statistical pattern recognition approach [2], each pattern is a collection of a number *C* of parameters, or features, extracted from the monitoring data and can be thought of as a point in a *C*-dimensional space. Feature selection is a very important step: the goal is to select a damage feature that allows pattern vectors associated with different structural states to occupy disjoint regions in the *C*-dimensional space, such that decision boundaries can be established to detect abnormal structural conditions [44]. This means that a good damage feature must be highly correlated with the severity of damage, and, ideally, lowly correlated to confounding effects, i.e., environmental and operational variations. Moreover, it is important to assess that little changes in the structural condition reflect into detectable changes in the damage feature.

Modal parameters are arguably among the most commonly adopted vibration-based damage features [45], mainly eigenfrequencies and mode shapes [46,47] (few successful examples about the use of modal damping as a damage feature can be found in the literature [48], mainly due to the complexity of the damping mechanism and the uncertainty associated with its estimate [49,50]). This paper focuses on eigenfrequencies, which can be easily estimated relying on few sensors and are usually less affected by experimental noise with respect to mode shapes [51]. The main drawback of adopting eigenfrequencies as damage features is related to the fact that they are highly sensitive to environmental variations [52]. Indeed, temperature variations can cause changes in eigenfrequency values which are greater than those caused by damage at an early stage. Moreover, usually only few low eigenfrequencies can be identified, which might not be sensitive enough to local damage (e.g., the presence of a crack), because local damage reflects on high vibration modes [2].

In the paper [22], the authors showed how a feature vector composed by the eigenfrequencies of multiple vibration modes of a tie-rod can be used to detect damage and in paper [23] the conclusions were validated in the presence of real damage. The multivariate feature vector $\underline{f}$ based on eigenfrequencies is defined as it follows:

(1) $$\underline{f}=\left[\begin{array}{c} f_{1}\\ f_{2}\\ \vdots \\ f_{m}\\ \vdots \\ f_{M} \end{array}\right]$$

where $f_{m}$ (with m=1,⋯,M) are the eigenfrequencies of *M* considered vibration modes.

The key point of the proposed approach is that eigenfrequencies are used to synthetically represent the state of the monitored tie-rod, since they are representative of the physical variables that mostly influence its dynamic behaviour (e.g., the axial load). Although these variables change due to environmental and operational conditions, variations associated with the confounding effects (e.g., temperature) give rise to different patterns with respect to those due to damage, in the multivariate feature space. As an example, the eigenfrequencies of the first three bending vertical modes of a healthy tie-rod are considered: a decrease of temperature would cause an increase in the values of all three the eigenfrequencies and the lower the vibration mode considered, the higher the effect [22]. If the temperature does not change but damage (e.g., a reduction of cross-section) is present at midspan, only the eigenfrequencies of the odd vibration modes would change (midspan is a vibration node for the even vibration modes) and the higher the vibration mode considered, the higher the effect [22]. The damage feature $\underline{f}$ can be adopted to exploit this observation: in the multivariate feature space defined by the eigenfrequencies of multiple vibration modes, patterns related to temperature variations differ from those related to damage. Thus, the damage feature $\underline{f}$ allows for the separation between damaged and undamaged states even in the presence of environmental and operational variations, and, for this reason, it will be adopted to define a novel damage index based on unsupervised learning data clustering, as it will be discussed in the next subsection.

### 2.3. Damage Indexes Based on Unsupervised Learning Approach

Once the damage feature is selected, a training phase is needed to learn the relationship between a pattern and the health condition of the monitored structure. Labelled data must be used for this scope, i.e., data for which the health state of the structure is known. If training data are available for all structural conditions (healthy as well as damaged), the problem is in the area of supervised learning. In the context of real operating structures, training data related to damage conditions are often not available. In this case, the problem is of unsupervised learning: the objective is learning the intrinsic relationship between the pattern and the normal structural condition only, defining a single class (undamaged state) and then test whether new data are still consistent with that class. According to this approach, damage detection can be framed as a problem of outlier detection (or novelty detection) or, alternatively, of unsupervised data clustering. These two approaches are compared in this work, where a novel damage index based on GMM is introduced. In more detail, the benchmark model will be the one based on the classical MSD, which is often adopted for unsupervised learning outlier detection, and it will be introduced in Section 2.3.1. The novel damage index will be introduced in Section 2.3.2, where the basics of GMM are also recalled.

#### 2.3.1. The Benchmark Approach Based on Outlier Detection

The outlier analysis is based on computing a discordancy measure between the damage feature calculated on new data and a reference training set of damage features calculated when the structure is in what is assumed to be a healthy condition. If the discordancy measure exceeds a threshold, an abnormal condition is detected. The case of interest for this work is the multivariate outlier detection, since $\underline{f}$ is a feature vector. In case the damage feature is multivariate, the discordancy measure is given by the MSD. For a generic multivariate feature vector ${\underline{f}}^{\mathrm{new}}$, its MSD from a generic matrix $\left[f\right]$ where every row contains an observation of the feature vector, can be calculated according to the following formula [53]:

(2) $$MSD\left({\underline{f}}^{\mathrm{new}},\left[f\right]\right)={\left({\underline{f}}^{\mathrm{new}}-{\underline{\mu }}_{f}\right)}^{T}{\left[\Sigma_{f}\right]}^{-1}\left({\underline{f}}^{\mathrm{new}}-{\underline{\mu }}_{f}\right)$$

where ${\underline{\mu }}_{f}$ and $\left[\Sigma_{f}\right]$ are the multivariate mean vector and the covariance matrix of $\left[f\right]$ respectively, and the suffix “−1” means the inverse.

In case the MSD is adopted, a damage index can be obtained calculating the MSD between the candidate feature vector ${\underline{f}}^{\mathrm{new}}$ and the baseline matrix ${\left[f\right]}^{\mathrm{base}}$ containing the normal condition features, i.e.,:

(3) $$DI=MSD({\underline{f}}^{\mathrm{new}},{\left[f\right]}^{\mathrm{base}})$$

Using the Gaussian assumption for the normal condition, the threshold can be calculated in terms of chi-squared-statistic [54] or adopting a numerical method. The latter approach is here adopted, based on the Monte Carlo method described in [53], and consisting in the following steps:

1. Populate a $\left(R\times C\right)$ matrix with randomly generated numbers from a zero-mean and unit-standard-deviation normal distribution, where *R* is the number of samples in the baseline matrix and *C* is the number of variables of the damage feature.
2. Calculate the MSD between every row of the matrix and the matrix itself and store the maximum distance.
3. Repeat the first two steps for a high number of trials (e.g., 1000) and store the resulting maxima in an array. The critical values for 1% or 5% tests of discordancy are given by the MSDs in the array above which 1% or 5% of the trials occurs, obtaining the so-called “inclusive threshold” $t_{\mathrm{inc}}$ (i.e., the presence of data coming from the damaged structure in the baseline set is admitted).
4. If the baseline set only includes data coming from the undamaged structure (as in the case considered in this work), the so-called exclusive threshold can be calculated according to the following expression [2]:
  (4) $$t_{\mathrm{exc}}=\frac{\left(R-1\right){\left(R+1\right)}^{2}t_{\mathrm{inc}}}{R\left(R^{2}-\left(R+1\right)t_{\mathrm{inc}}\right)}$$

MSD-based damage detection is one of the most popular methods in SHM due to its ease of use and computational efficiency [55,56]. One of the major advantages of the MSD is that it can be used to filter out the environmental effects on damage features, provided that the baseline set contains a wide range of environmental conditions [57].

#### 2.3.2. The New Approach Based on Unsupervised Data Clustering

A new approach to damage detection in tie-rods is proposed in this section, by framing the problem as unsupervised data clustering. As mentioned above, a damage feature can be thought of as a point in a *C*-dimensional space. If an appropriate damage feature is selected, in a way that feature vectors associated with different structural states occupy disjoint regions in the *C*-dimensional domain, a data clustering approach can be adopted to carry out damage detection. The main premise is that there is a migration of groups (or clusters) of feature vectors as damage occurs to the structure. In this work, for the first time in the literature of tie-rod damage detection, the deterministic physics-based behaviour of tie-rod natural frequencies is exploited: a new multivariate domain is defined where the existence of clusters of damage-related data can be automatically spotted, setting the stage for unsupervised data clustering algorithms to be successfully adopted. Before going into details of the specific case-study, a brief recall of the theory related to the adopted algorithm (i.e., GMM) is presented.

Unsupervised data clustering can be carried out according to different approaches, e.g., statistical, neural and syntactic methods [58]. As all SHM problems are subject to various degrees of uncertainty, the statistical approaches appear to stand out as a natural choice for damage detection in real operating structures. Finite mixtures allow addressing the problem in a probabilistic way, since they inherently account for the uncertain nature of the problem. According to these approaches, every observation of the damage feature is considered as generated by one among a set of alternative random sources. Inferring the parameters of these sources allows one associating a given observation to the probability to have been generated by one specific source. In this way, data can be automatically clustered [59].

A GMM is a parametric probability density function made by a weighted sum of a number *K* of Gaussian densities (as it will be shown in the following, this probability density function is particularly suitable to fit the probability density function of the adopted damage feature). In the following, the generic matrix $\left[x\right]$ is considered, which is a random sample set composed by a number *R* of independent random samples. A generic *C*-dimensional sample in the sample set is indicated by ${\underline{x}}_{r}$, with r=1,⋯,R; ${\underline{x}}_{r}$ is a row of the matrix $\left[x\right]$ and it is one observation of the feature vector. By assuming that ${\underline{x}}_{r}$ follows a GMM, the probability density function can be written as a mixture of Gaussian distributions according to the following expression:

(5) $$p\left({\underline{x}}_{r}|\lambda \right)=\sum_{i=1}^{K}\theta_{i}N_{i}\left({\underline{x}}_{r}|{\underline{\mu }}_{i},{\left[\Sigma \right]}_{i}\right)$$

where $\theta_{i}$ are the weights and each $N_{i}\left({\underline{x}}_{r}|{\underline{\mu }}_{i},{\left[\Sigma \right]}_{i}\right)$ is a *C*-variate Gaussian density defined as it follows:

(6) $$N_{i}\left({\underline{x}}_{r}|{\underline{\mu }}_{i},{\left[\Sigma \right]}_{i}\right)=\frac{1}{{\left(2\pi \right)}^{C/2}{\left|{\left[\Sigma \right]}_{i}\right|}^{1/2}}\exp\left\{-\frac{1}{2}{\left({\underline{x}}_{r}-{\underline{\mu }}_{i}\right)}^{T}{\left[\Sigma \right]}_{i}^{-1}\left({\underline{x}}_{r}-{\underline{\mu }}_{i}\right)\right\}$$

fully characterized by the *i*-th mean vector ${\underline{\mu }}_{i}$ and *i*-th covariance matrix ${\left[\Sigma \right]}_{i}$. The weights $\theta_{i}$ must satisfy the conditions $\theta_{i}\geq 0$ for i=1,⋯,K and $\sum_{i=1}^{K}\theta_{i}=1$. For notation convenience, all the parameters that specify the mixture are collected in the set of parameters λ, defined as it follows:

(7) $$\lambda \equiv \left\{\theta_{1},{\underline{\mu }}_{1},{\left[\Sigma \right]}_{1},\cdots ,\theta_{K},{\underline{\mu }}_{K},{\left[\Sigma \right]}_{K}\right\}$$

In order to adopt GMM for unsupervised data clustering, a set of parameters λ must be estimated so that the probability density function of the GMM best fits the distribution of the samples in the sample set $\left[x\right]$. The maximum likelihood estimate of the parameters can be obtained by maximizing the following expression:

(8) $$L\left(\lambda \right)=p\left(\left[x\right]|\lambda \right)=\prod_{r=1}^{R}p\left({\underline{x}}_{r}|\lambda \right)$$

which is the likelihood of the GMM, given a population containing a number *R* of feature vectors (a $\left(R\times C\right)$ matrix $\left[x\right]$), assuming independence among different observations. The maximization of the term in Equation (8) cannot be carried out analytically and, for this reason, an iterative procedure called expectation maximization (EM) algorithm [60] (see Appendix B) is adopted.

Now that the basics of GMM have been recalled for a general case, the proposed damage index is discussed for the specific application. First, the assumed hypothesis is that squared eigenfrequencies of different vibration modes are related to each other with a linear relationship, if the axial load is the only changing variable. If a number *M* of vibration modes is considered, the baseline matrix set ${\left[f\right]}^{\mathrm{base}}$ matrix contains a number *M* of columns. Each column, represents the trend in time of the *m*-th eigenfrequency, with m=1,⋯,M (m=1 indicates the lowest considered vibration mode, not necessarily the first). If all the elements in ${\left[f\right]}^{\mathrm{base}}$ are squared, each column represents the trend in time of the squared *m*-th eigenfrequency, and it will be indicated as ${\underline{s}}_{m}^{\mathrm{base}}$.

A number M−1 of couples can be defined by choosing the lowest squared eigenfrequency ${\underline{s}}_{1}^{\mathrm{base}}$ and one of the other ${\underline{s}}_{m}^{\mathrm{base}}$ squared eigenfrequency, with 1<m≤M. The coefficients of the linear regression $a_{1m}$ and $b_{1m}$ can be estimated from the least squares solution of the linear problem defined in the following equation:

(9) $${\underline{s}}_{m}^{\mathrm{base}}=a_{1m}\cdot {\underline{s}}_{1}^{\mathrm{base}}+b_{1m}\cdot \underline{1}$$

where $\underline{1}$ is an all-ones column vector of the same size as ${\underline{s}}_{m}^{\mathrm{base}}$ or ${\underline{s}}_{1}^{\mathrm{base}}$. Once the coefficients $a_{1m}$ and $b_{1m}$ are known, the residuals of the linear regression can be calculated according to next equation:

(10) $${\underline{\varepsilon }}_{1m}^{\mathrm{base}}={\underline{s}}_{m}^{\mathrm{base}}-a_{1m}\cdot {\underline{s}}_{1}^{\mathrm{base}}-b_{1m}\cdot \underline{1}$$

which, if the hypothesis of linear relationship is verified, are Gaussian distributed.

Starting from a baseline matrix ${\left[f\right]}^{\mathrm{base}}$ with *M* columns, solving the linear problem of Equation (9) and calculating the residuals according to Equation (10) for all the M−1 couples of columns, a matrix ${\left[\varepsilon \right]}^{\mathrm{base}}$ can be obtained with M−1 columns.

The same procedure can be applied to the data contained in a matrix ${\left[f\right]}^{\mathrm{new}}$, containing the eigenfrequencies of the *M* considered vibration modes, in a period different from that of the baseline. In this case, the coefficients $a_{1m}$ and $b_{1m}$, which are those estimated by considering the baseline set, will be used to calculate the residuals, i.e.,:

(11) $${\underline{\varepsilon }}_{1m}^{\mathrm{new}}={\underline{s}}_{m}^{\mathrm{new}}-a_{1m}\cdot {\underline{s}}_{1}^{\mathrm{new}}-b_{1m}\cdot \underline{1}$$

finally obtaining a matrix ${\left[\varepsilon \right]}^{\mathrm{new}}$.

The residuals calculated on the baseline set and those calculated on the new data can be put together in the same matrix $\left[\varepsilon \right]$, according to the next expression:

(12) $$\left[\varepsilon \right]=\left[\begin{array}{c} {\left[\varepsilon \right]}^{\mathrm{base}}\\ {\left[\varepsilon \right]}^{\mathrm{new}} \end{array}\right]$$

which is a population containing a number *R* of observations of the *C*-dimensional feature vector ${\underline{\varepsilon }}_{r}$, where C=M−1.

The problem of damage detection can now be framed under a probabilistic point of view, studying the probability density function of the generic *C*-dimensional sample ${\underline{\varepsilon }}_{r}$. When no damage is present, the hypothesis is that the probability density function of ${\underline{\varepsilon }}_{r}$ follows a single *C*-variate Gaussian density, i.e.,

(13) $$p\left({\underline{\varepsilon }}_{r}|\gamma \right)=N\left({\underline{\varepsilon }}_{r}|\underline{\mu },\left[\Sigma \right]\right)$$

where:

(14) $$\gamma \equiv \left\{\underline{\mu },\left[\Sigma \right]\right\}$$

are the parameters that maximize the likelihood of the model:

(15) $$L\left(\gamma \right)=p\left(\left[\varepsilon \right]|\gamma \right)=\prod_{r=1}^{R}p\left({\underline{\varepsilon }}_{r}|\gamma \right)$$

When the set ${\left[\varepsilon \right]}^{\mathrm{new}}$ contains data coming from the damaged structure, the hypothesis is that the probability density function of ${\underline{\varepsilon }}_{r}$ follows a GMM composed by a mixture of two *C*-variate Gaussian densities: one that best fits the baseline residuals ${\left[\varepsilon \right]}^{\mathrm{base}}$ and the other that best fits the damaged set ${\left[\varepsilon \right]}^{\mathrm{new}}$. According to Equation (5), ${\underline{\varepsilon }}_{r}$ has a probability density function that can be written as:

(16) $$p\left({\underline{\varepsilon }}_{r}|\lambda \right)=\sum_{i=1}^{2}\theta_{i}N_{i}\left({\underline{\varepsilon }}_{r}|{\underline{\mu }}_{i},{\left[\Sigma \right]}_{i}\right)$$

fully characterized by the parameters

(17) $$\lambda \equiv \left\{\theta_{1},{\underline{\mu }}_{1},{\left[\Sigma \right]}_{1},\theta_{2},{\underline{\mu }}_{2},{\left[\Sigma \right]}_{2}\right\}$$

which can be estimated adopting the EM algorithm. The parameters contained in λ maximize the likelihood of the GMM made by two Gaussian distributions given the population of features $\left[\varepsilon \right]$, which can be calculated as:

(18) $$L\left(\lambda \right)=p\left(\left[\varepsilon \right]|\lambda \right)=\prod_{r=1}^{R}p\left({\underline{\varepsilon }}_{r}|\lambda \right)$$

The validity of the two hypotheses (single versus double *C*-variate Gaussian distribution) can be assessed by comparing $L\left(\gamma \right)$ and $L\left(\lambda \right)$. To do so, the parameters γ and λ, estimated adopting the EM algorithm, are used to calculate $L\left(\gamma \right)$ and $L\left(\lambda \right)$ given the data $\left[\varepsilon \right]$ through Equations (15) and (18), respectively. For numerical convenience, the logarithm is used to transform products of potentially small likelihoods into a sum of logarithmic values, which are easier to distinguish from 0, in computation. Furthermore, optimization algorithms often search for the minima of functions; thus, the negative log-likelihood values will be adopted hereinafter, defined by the following Equations (19) and (20).

(19) $$L_{1}=-\log\left(L\left(\gamma \right)\right)$$

(20) $$L_{2}=-\log\left(L\left(\lambda \right)\right)$$

The general idea is that if $L\left(\lambda \right)$ is significantly higher than $L\left(\gamma \right)$ or, equivalently, $L_{2}$ is significantly lower than $L_{1}$, there is a high likelihood of damage. To show the validity of the approach, data coming from the experimental set-up are now discussed. In the following, a real damage condition is considered, which is that associated with the effects of general corrosion close to the constraints.

In this case, three eigenfrequencies are considered, which are those associated with the bending vertical modes number 4, 5 and 6. The use of three eigenfrequencies (M=3) returns a matrix of residuals with two columns (C=M−1=2), which allows for a convenient representation adopting two-dimensional scatterplots.

**Figure 2:**
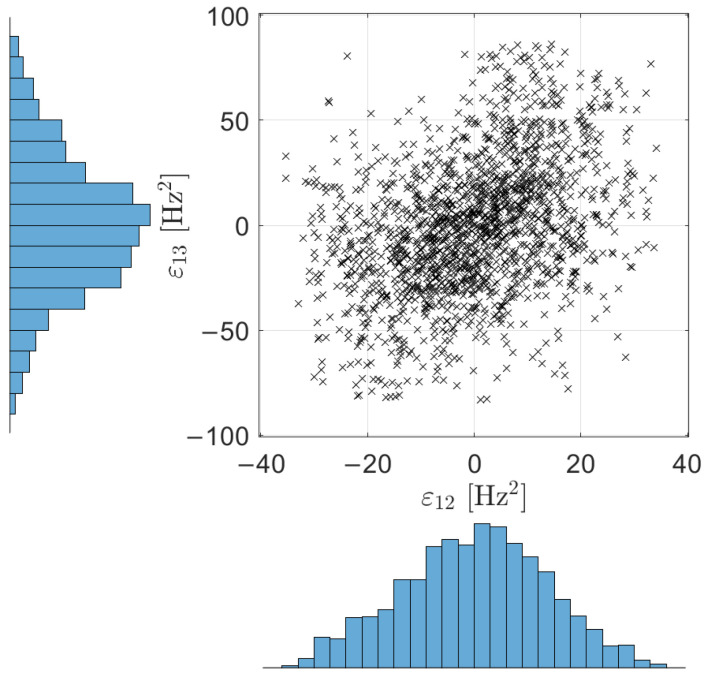
Scatterplot of the data contained in ${\left[\varepsilon \right]}^{\mathrm{base}}$ for the baseline period and histograms of ${\underline{\varepsilon }}_{12}^{\mathrm{base}}$ and ${\underline{\varepsilon }}_{13}^{\mathrm{base}}$.

First, the set of ${\left[\varepsilon \right]}^{\mathrm{base}}$ is considered, with data referring to 1646 hours collected in the periods 19 December 2019 to 3 January 2020, and 22 April 2020 to 14 August 2020, after the adoption of the data cleansing algorithm described in [23]. A scatterplot of ${\underline{\varepsilon }}_{12}^{\mathrm{base}}$ versus ${\underline{\varepsilon }}_{13}^{\mathrm{base}}$ is reported in Figure 2. Below the x-axis and to the left side of the y-axis, the histograms of the data of ${\underline{\varepsilon }}_{12}^{\mathrm{base}}$ and ${\underline{\varepsilon }}_{13}^{\mathrm{base}}$ are reported, respectively.

**Figure 3:**
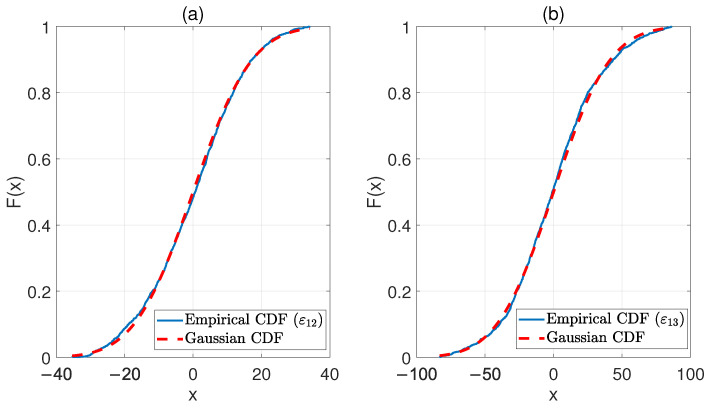
Comparison between the Empirical CDF and the Gaussian CDF, using the data in ${\underline{\varepsilon }}_{12}^{\mathrm{base}}$ (**a**) and ${\underline{\varepsilon }}_{13}^{\mathrm{base}}$ (**b**).

The histograms of Figure 2 suggest that the residuals in ${\underline{\varepsilon }}_{12}$ and ${\underline{\varepsilon }}_{13}$ are Gaussian distributed, as confirmed by a visual comparison between the empirical cumulative distribution function (CDF) and the Gaussian CDF for $\varepsilon_{12}$ and $\varepsilon_{13}$, reported in Figure 3a,b, respectively. Thus, the data set contained in ${\left[\varepsilon \right]}^{\mathrm{base}}$ is characterized by a bi-variate Gaussian probability density function.

**Figure 4:**
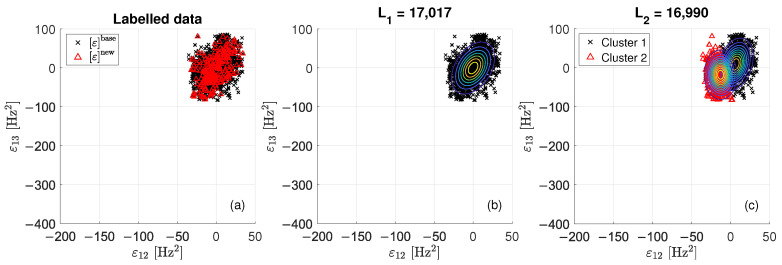
Scatterplots of ${\left[\varepsilon \right]}^{\mathrm{base}}$ and ${\left[\varepsilon \right]}^{\mathrm{new}}$ when no damage is present: labelled data (**a**), single bi-variate Gaussian distribution (**b**) and mixture of two bi-variate Gaussian distributions (**c**).

**Figure 5:**
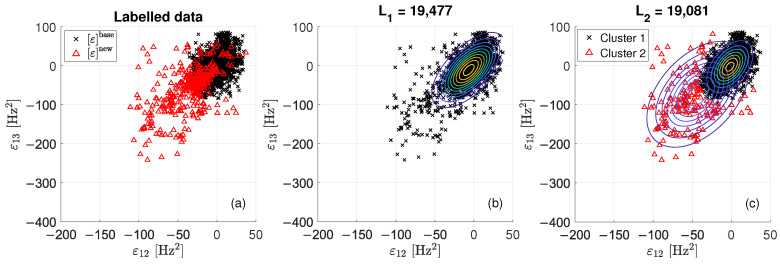
Scatterplots of ${\left[\varepsilon \right]}^{\mathrm{base}}$ and ${\left[\varepsilon \right]}^{\mathrm{new}}$ when damage at an early stage is present: labelled data (**a**), single bi-variate Gaussian distribution (**b**) and mixture of two bi-variate Gaussian distributions (**c**).

**Figure 6:**
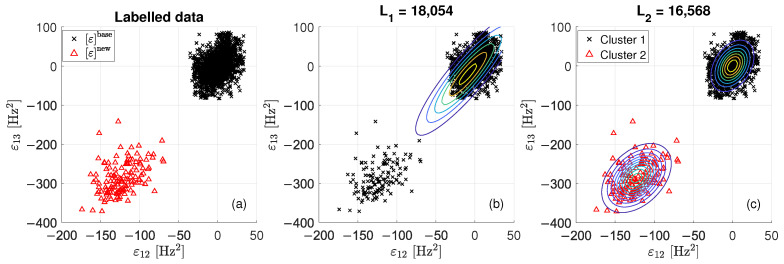
Scatterplots of ${\left[\varepsilon \right]}^{\mathrm{base}}$ and ${\left[\varepsilon \right]}^{\mathrm{new}}$ when a severe damage condition is present: labelled data (**a**), single bi-variate Gaussian distribution (**b**) and mixture of two bi-variate Gaussian distributions (**c**).

The proposed damage detection strategy is explained through three figures (Figure 4, Figure 5 and Figure 6), which are representative of three different tie-rod conditions. Each of the three figures is composed of three sub-plots, “a”, “b” and “c”. The sub-plot “a” shows the data in the matrix $\left[\varepsilon \right]$, adopting black crosses and red triangles to indicate data belonging to ${\left[\varepsilon \right]}^{\mathrm{base}}$ and ${\left[\varepsilon \right]}^{\mathrm{new}}$, respectively. The sub-plot “b” shows the probability density function of a single bi-variate Gaussian distribution, adopting contour lines to help visualizing the single centroid of the distribution; the value of $L_{1}$ is reported above the figure (in the following, $L_{1}$ and $L_{2}$ will be used to define a damage index). The sub-plot “c” shows the probability density function resulted from the adoption of the EM algorithm, again adopting contour lines to help visualizing the centroids of the two bi-variate Gaussian distributions composing the mixture. Data are divided into two clusters: data belonging to “cluster 1” are represented with black crosses, while data belonging to “cluster 2” are represented with red triangles. The value for $L_{2}$ is reported above the figure.

When data contained in ${\left[\varepsilon \right]}^{\mathrm{new}}$ are associated with a period when no damage is present (see Figure 4), the hypothesis of a single bi-variate Gaussian distribution that was observed for ${\left[\varepsilon \right]}^{\mathrm{base}}$ is still valid. Indeed, data of ${\left[\varepsilon \right]}^{\mathrm{new}}$ seems to be generated by the same probability density function of ${\left[\varepsilon \right]}^{\mathrm{base}}$, as confirmed by the fact that red triangles and black crosses are overlapped and mixed in Figure 4a. By comparing the probability density functions of the single bi-variate Gaussian distribution (Figure 4b) with that of the mixture (Figure 4c), the clustering solution proposed in Figure 4c seems to be due to over fitting. Moreover, it is worth noting that the value of $L_{2}$ is almost the same of that of $L_{1}$ ($L_{2}$ is 0.16% smaller than $L_{1}$).

Data presented in Figure 5 are associated with the presence of damage at an early stage. By a visual check of data in Figure 5a, the hypothesis that data of ${\left[\varepsilon \right]}^{\mathrm{new}}$ belong to a different cluster with respect to that of the baseline data seems more likely. The EM algorithm proposes the clustering reported in Figure 5c. The value of $L_{2}$ is a 2% smaller than that of $L_{1}$.

When data in ${\left[\varepsilon \right]}^{\mathrm{new}}$ are associated with a period when a severe state of damage is present, points of ${\left[\varepsilon \right]}^{\mathrm{new}}$ and ${\left[\varepsilon \right]}^{\mathrm{base}}$ are disjointed (see Figure 6a). The EM converges to the solution presented in Figure 6c, showing that data in ${\left[\varepsilon \right]}^{\mathrm{new}}$ and ${\left[\varepsilon \right]}^{\mathrm{base}}$ belong to two different clusters. In this case, the value of $L_{2}$ is a 9% smaller than that of $L_{1}$.

The three cases discussed here suggest that the strategy can be automatized by using a measure of the likelihood increment obtained by adopting a mixture of bi-variate Gaussian distributions in place of a single bi-variate Gaussian distribution to model the probability density function of $\left[\varepsilon \right]$.

According to this idea, in a more general framework, the evaluation of the state of the structure can be carried out by assembling a matrix $\left[\varepsilon \right]$, running the EM algorithm to fit a GMM with two components and calculating the values of $L_{1}$ and $L_{2}$. A novel damage index is defined, according to the following Equation (21):

(21) $$LI=\frac{L_{1}-L_{2}}{L_{2}}$$

**Figure 7:**
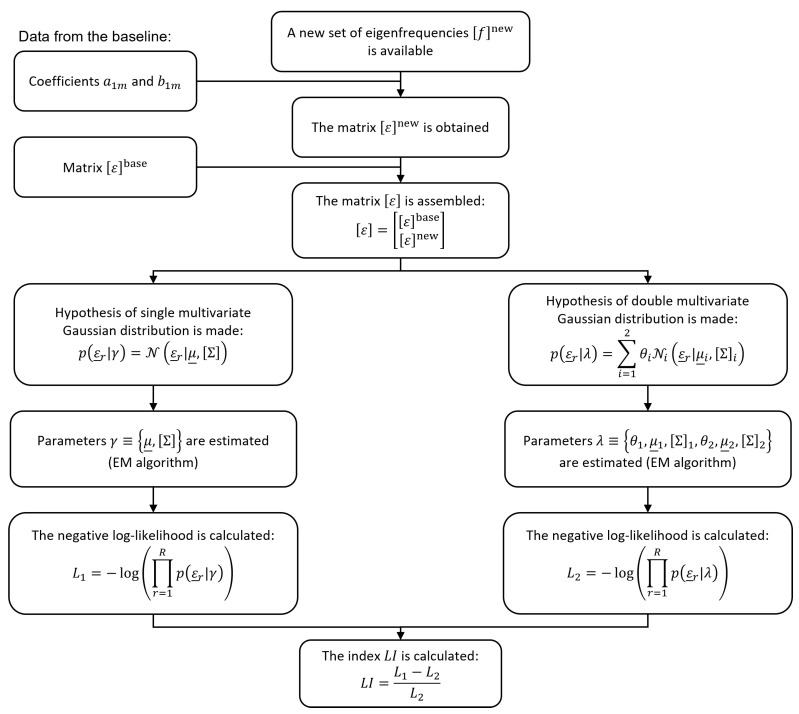
Flowchart of the procedure to calculate the damage index LI.

LI can be adopted to quantify the likelihood increment obtained by passing from a single *C*-variate Gaussian distribution to a mixture of two *C*-variate Gaussian distributions (the procedure to calculate the index LI is described in the flowchart of Figure 7). The difference between $L_{1}$ and $L_{2}$ is normalized by dividing its value by $L_{2}$, which is that associated with the model that is always able to best fit the underlying probability density function and, thus, set as reference. Indeed, the mixture can also approximate the single bi-variate Gaussian distribution (see the contour lines of Figure 4c as an example). On the contrary, the model based on a single *C*-variate Gaussian distribution fails to describe the probability density function of $\left[\varepsilon \right]$, when more than one cluster exists in the data (see the contour lines of Figure 6b as an example).

In the following section, DI (see Equation (3) in Section 2.3.1) and LI will be compared, to assess which approach can allow for an earlier damage detection, considering long term monitoring data and the effects of real damage on a tie-rod.

## 3. Results

The two damage indexes are compared considering two case studies. The first is the most challenging one since damage occurs close to the constraints, at a distance equal to $\frac{1}{10}W$ (*W* is the beam free length) from one of the fixed ends of the tie-rod. Indeed, when damage is close to the constraints, the eigenfrequencies are less sensitive, as already observed in [22]. The damage scenario is associated with the real effects of the corrosive attack induced on the tie-rod surface, that affected a length of the tie-rod of approximately 5 cm. Some pictures of different stages of the corrosion process are reported in Table 1, and they are labelled with letters A1, B1, C1 and D1. To provide not just a qualitative picture, but also a quantitative one, the different cross-section reductions Δh are reported (e.g., Δh=6% means a reduction of 6% of the cross-section height, measured at the centre of the corroded area).

**Figure 8:**
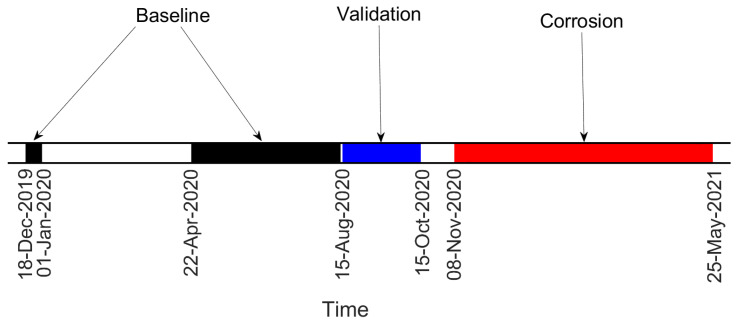
Timeline of the test on the tie-rod, corroded at a distance of $\frac{1}{10}W$ from the constraints.

**Figure 9:**
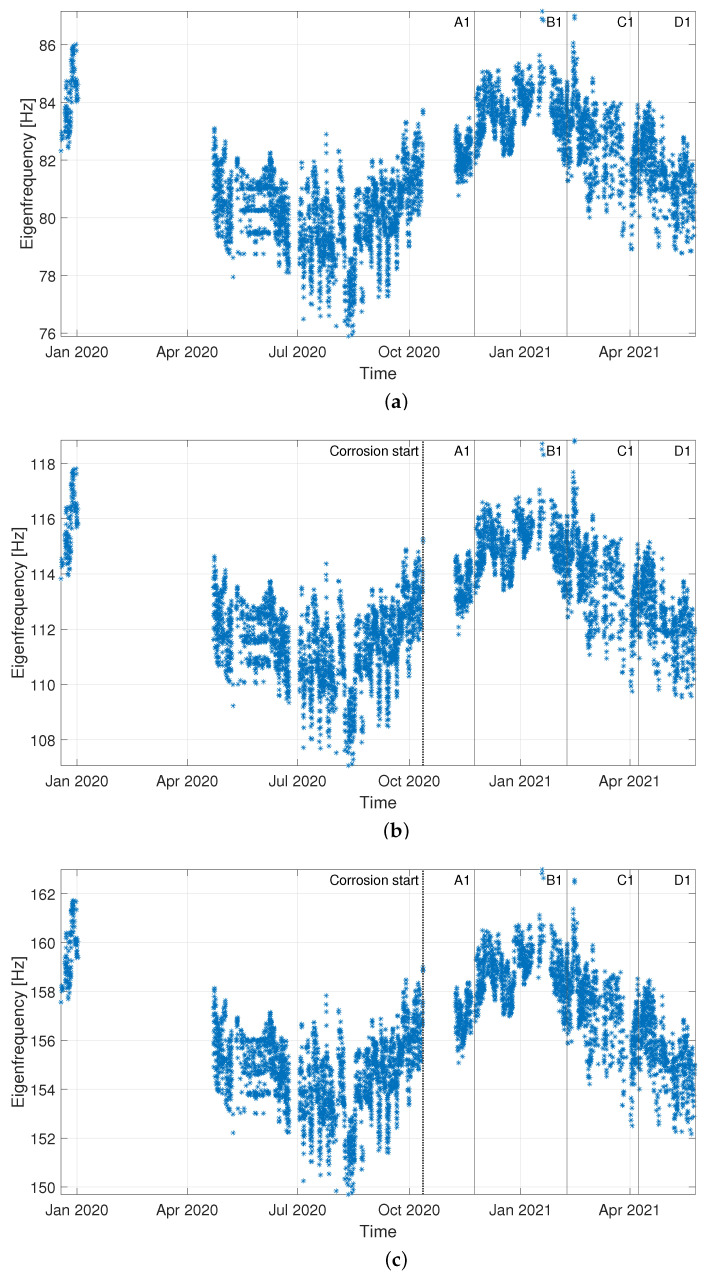
Case 1: time trends of the considered eigenfrequencies, i.e., those of the fourth (**a**), fifth (**b**) and sixth (**c**) bending vibration modes in the vertical plane.

Data are divided into “Baseline”, “Validation” (i.e., data not included in the baseline set, but still related to a condition when no damage is present) and “Corrosion” sets, according to the timeline of Figure 8. For both damage indexes, the results presented are obtained considering the eigenfrequencies associated with three vibration modes, more specifically those of the fourth, fifth and sixth bending vibration modes in the vertical plane (see Figure 9). It is recalled that the eigenfrequencies are obtained by adopting operational modal analysis, thus exploiting the excitation coming from the operational environment (see Section 2.1).

**Figure 10:**
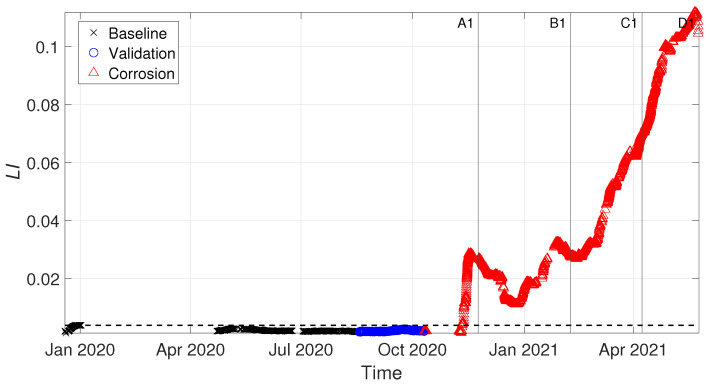
Damage index LI for tie-rod 1.

The damage index LI is evaluated every time a new set of frequencies is available, assembling the matrix composed by the last month of data ${\left[\varepsilon \right]}^{\mathrm{new}}$ with the entire baseline set ${\left[\varepsilon \right]}^{\mathrm{base}}$ (see Section 2.3.2). The results are reported Figure 10, where vertical lines are labelled according to Table 1, to point at the different tie-rod conditions. The damage threshold is indicated with a black-dashed horizontal line, and it indicates the 99th percentile of the baseline set, considering that no damage was present herein.

**Figure 11:**
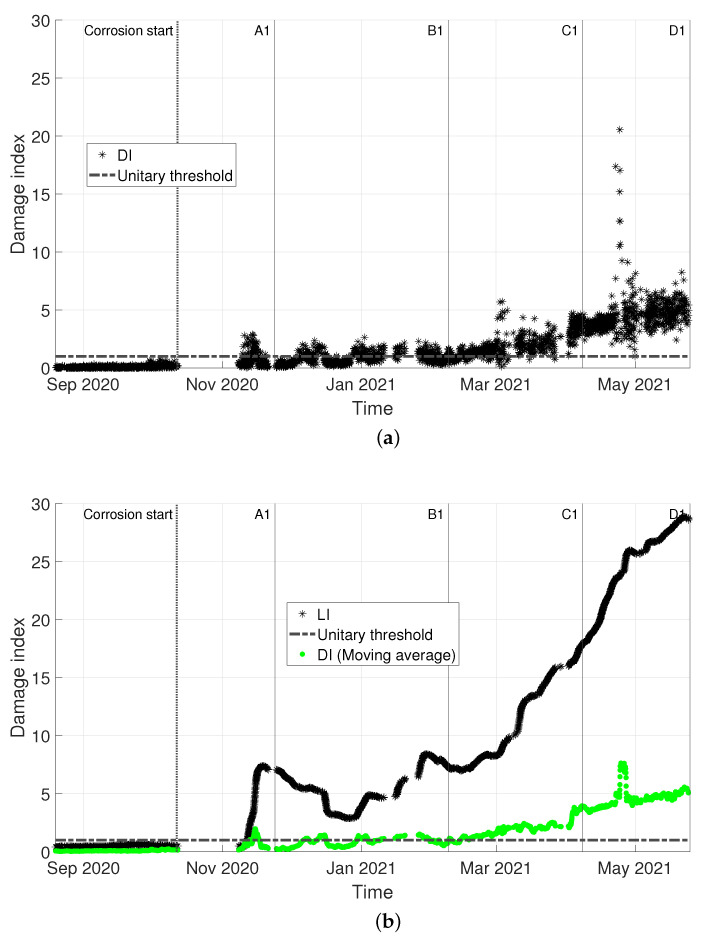
Case 1: comparison of the two automatic damage detection approaches on the same data. (**a**) MSD-based damage index DI, normalized on its damage threshold. (**b**) GMM-based damage index LI, normalized on its damage threshold (black asterisks) and MSD-based damage index DI obtained through a moving average process (green points).

In Figure 11, a comparison with the benchmark approach (DI, based on the MSD) is shown. To allow for a direct comparison of the two trends, both DI and LI are normalized to the respective damage detection threshold and they are shown in Figure 9a and Figure 9b, respectively. In more detail, the damage threshold for DI is calculated according to the procedure explained in Section 2.3.1, adopting Equation (4), where $t_{\mathrm{inc}}$ is obtained considering the critical value for 1% test of discordancy. For both LI and DI, black asterisks are used to represent the damage index; due to the scatter of DI, significantly higher than that of LI, also the trend obtained with a moving average (window of one day, overlap 1 hour) is reported with green points in Figure 9a, to allow for a clearer visualization and a direct comparison of the two trends. A zoom is made on the data sets “Validation” and “Corrosion”: a black-dashed vertical line indicates the start of the corrosion attack and, again, vertical lines labelled according to Table 1 are used to point at different tie-rod conditions. A dot-dashed-black horizontal line is used to indicate the unitary threshold.

The results related to another case study are reported in the following. In this second example, a different specimen is considered, where the corrosive attack was induced at a distance equal to $\frac{5}{8}W$ from one of the fixed ends. As for the previous case, different stages of the deteriorative process are reported in Table 2, together with the labels and cross-section reductions.

**Figure 12:**
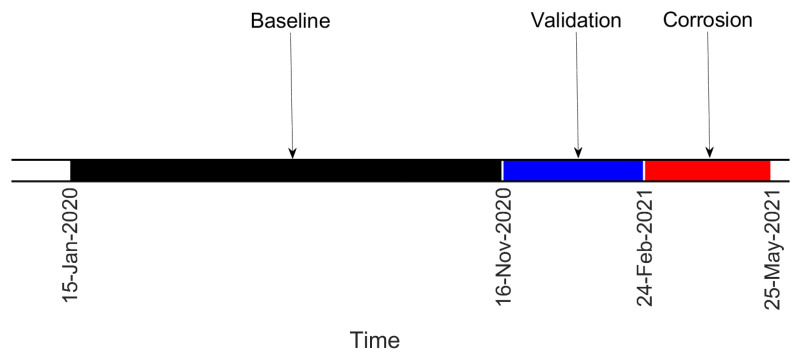
Timeline of the test on the tie-rod, corroded at a distance of $\frac{5}{8}W$ from the constraints.

**Figure 13:**
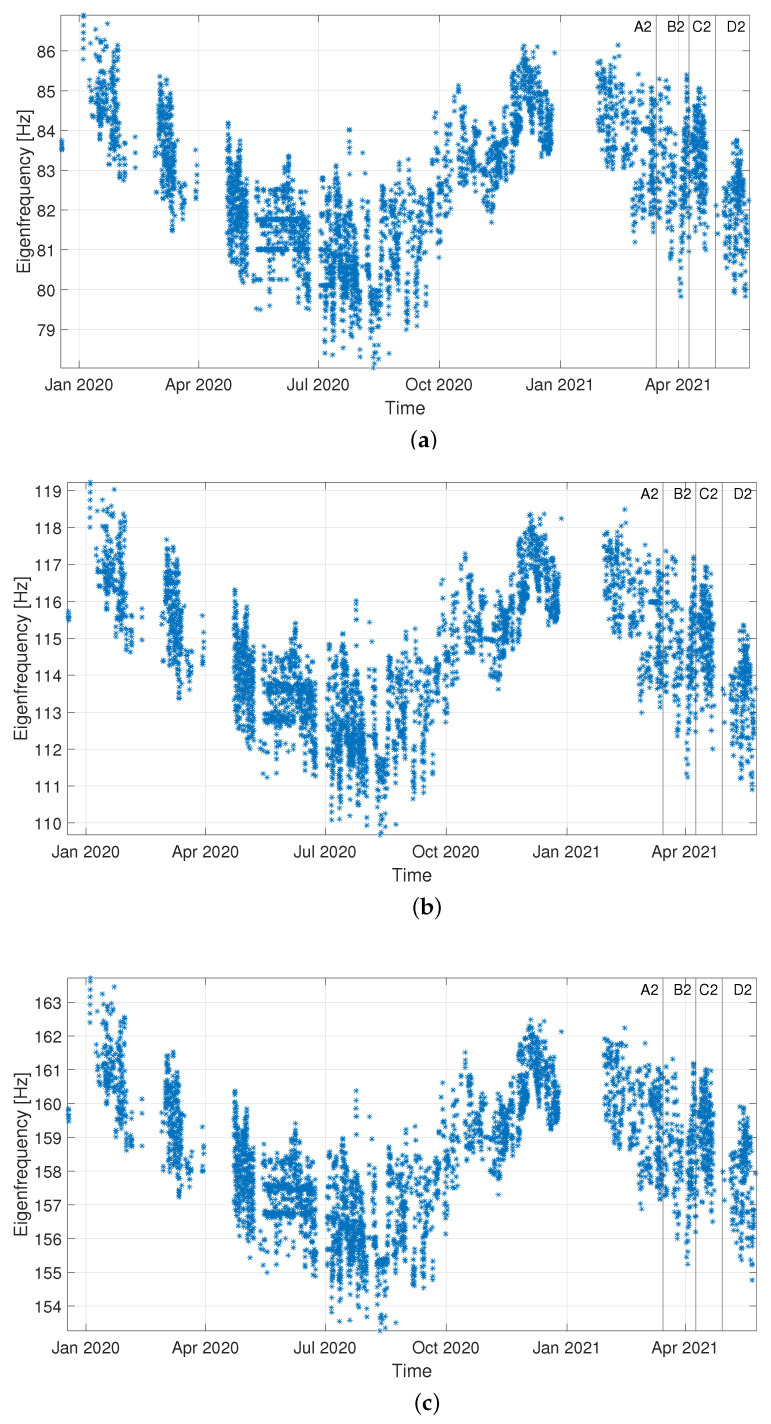
Case 2: time trends of the considered eigenfrequencies, i.e., those of the fourth (**a**), fifth (**b**) and sixth (**c**) bending vibration modes in the vertical plane.

**Figure 14:**
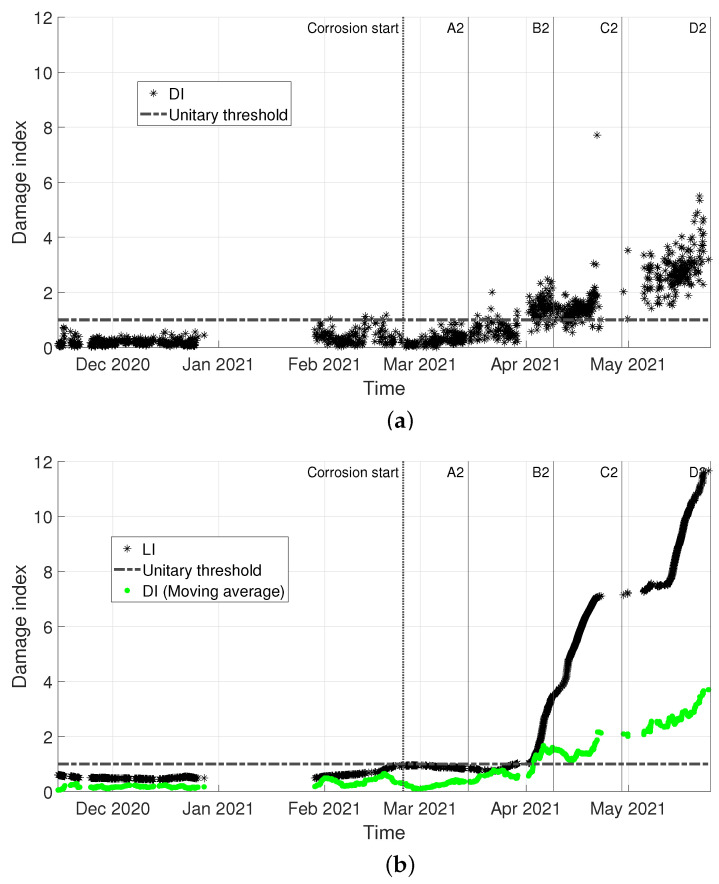
Case 2: comparison of the two automatic damage detection approaches on the same data. (**a**) MSD-based damage index DI, normalized on its damage threshold. (**b**) GMM-based damage index LI, normalized on its damage threshold (black asterisks) and MSD-based damage index DI obtained through a moving average process (green points).

Data are divided into “Baseline”, “Validation” and “Corrosion” sets, according to the timeline of Figure 12. For both damage indexes, the results presented are obtained considering the eigenfrequencies associated with the fourth, fifth and sixth bending vibration modes in the vertical plane (see Figure 13). The comparison between DI and LI, both normalized to the respective damage detection threshold, is shown in Figure 14.

## 4. Discussion

A first consideration that is worth mentioning is related to the eigenfrequency trends reported in Figure 9 and Figure 13: a high variability can be observed, characterized by both daily and long-term trends related to temperature conditions. The magnitude of these fluctuations does not allow spotting the existence of damage, even when the eigenfrequency associated with the highest vibration mode (i.e., the sixth vibration mode) is considered. Indeed, even if the final stage of the corrosion process is considered (condition D1 in Figure 13c and condition D2 in Figure 9c), the eigenfrequency values are compatible with those observed in the previous year and they seem to follow the seasonal trend.

The effectiveness of the proposed approach is shown by the trend of LI in Figure 10: as it is possible to see, LI is exceeding the damage threshold when damage is present (red triangles in Figure 10). Damage is detected way before when the tie-rod is in condition B1 (see Table 1), which is remarkable considering that the corroded portion of the tie-rod is close to the fixed end. Furthermore, the blue circles associated with the validation set remain below the threshold, and this is consistent with the fact that the tie-rod is still in the same condition of the baseline set.

Coming to the comparison with the benchmark model, the damage index LI, based on the GMM, outperforms DI, based on the MSD, as it is shown in Figure 11. Indeed, DI is characterized by a higher variability (compare the black asterisks in Figure 11a with those of Figure 11b) which causes the damage index to be scattered around the damage detection threshold for a long time before it remains steadily above the threshold (approximately, between conditions C1 and D1). If the averaged trend of DI (green points in Figure 11b) is compared with LI (black asterisks in Figure 11b), it is clear how the novel GMM-based damage index has a higher sensitivity to damage, potentially allowing for a prompter damage detection. LI shows a clear trend that points out the presence of damage and its evolution over time, thanks to a higher robustness to the effects of environmental and operational variations.

The same remarks can be made regarding the second example here discussed, i.e., damage farther from the constraints. The adoption of a multivariate damage feature based on tie-rod eigenfrequencies allows spotting the presence of damage, with both DI and LI that cross the respective damage detection threshold, in Figure 14a and Figure 14b, respectively. LI is clearly detecting damage when the tie-rod is not yet in condition B2. It is worth noting that, at this stage, the damage effects are barely noticeable through a visual inspection (see Table 2). Once again, DI detects damage later than LI, staying constantly above the damage detection threshold approximately between conditions C2 and D2 (see Figure 14a).

In this second case study, the different performances between the two damage indexes can be clearly highlighted. First, if the averaged time-trend of DI is considered (green points in Figure 14b), fluctuations can be detected, while the time-trend of LI (black asterisks in Figure 14b) shows a more stable trend, again proving that the GMM-based damage index is more robust to environmental variations than the MSD-based one. Second, regarding the comparison between the trend of DI with no moving average and LI, DI is more sensitive to noise in the data than LI. If the black asterisks in Figure 14a are considered, the scatter of the results is partially associated with the uncertainty related to the modal identification (e.g., errors due to a poor signal-to-noise ratio of the original vibration data), which plays a significant role when the MSD is calculated between a single observation of the damage feature and the baseline set. On the contrary, when LI is estimated, the uncertainty is actually modelled by the GMM: the uncertainty in the data causes the spread of the damage features in the multivariate feature space (e.g., how wide the clouds of Figure 6 are), but does not reflect on the scatter of LI. Indeed, LI relies on the validity of the hypothesis related to the underlying probability density function, which significantly changes only when the deterministic effect of damage causes a migration of the centroid of the population of new data from the region occupied by the baseline data. So, even if a lower noise in the data would make it easier to distinguish different clusters, thus increasing the sensitivity of the method, the index LI is much more robust to noise in the data than DI.

To resume, the main advantages of the adoption of the GMM-based strategy are related to a higher sensitivity to damage and a lower uncertainty associated with the results with respect to the MSD-based strategy, as emerged from the experimental campaign. These characteristics are crucial when the damage is characterized by a progressive evolution in time, as in the case of corrosion. On the other hand, the main advantage of the MSD-based strategy with respect to the the GMM-based one is that it is potentially prompter in detecting fast events (e.g., a sudden failure of the constraint system). Indeed, if a single observation of the multivariate damage feature related to a fast damage event is considered, its effect can cause a high MSD from the baseline set which can immediately make DI cross the detection threshold. However, this could not be enough to cause a significant migration of the centroid of the population of new data with respect to the baseline cluster, until a sufficient number of damage-related observations is available, causing a delay between the occurrence of the failure and the moment when LI exceeds the threshold.

## 5. Conclusions

This paper presented a novel damage index for automatic damage detection in operating tie-rods, based on unsupervised learning data clustering through GMM. The damage index was compared with a benchmark one, estimated through one of the most adopted multivariate metrics for outlier detection: the MSD. Both damage indexes are calculated on the same database containing multiple observations of the feature vector composed by tie-rod eigenfrequencies. Although the MSD is effective in detecting damage, as already shown by the authors in previous works, the novel damage index based on GMM proved to be affected by a significantly lower scatter and to provide a higher sensitivity, which allows for an earlier damage detection. Indeed, the MSD considers each new observation of the damage feature against the entire reference population; thus, the results are characterized by a scatter which is mainly related to the variability of each identification of the damage feature. Consequently, the scatter is higher than that obtained with the GMM-based approach, where a population of data is tested against the baseline population. The GMM-based strategy, which can spot the onset of new clusters in the data in a completely unsupervised way, proved to be suitable to detect evolutive deteriorative phenomena, such as corrosion, outperforming the MSD-based approach. On the other hand, for other types of failure associated with faster phenomena (e.g., a sudden failure of the constraint system), the MSD-based approach should be more suitable to detect abrupt changes in the structural condition. A further development of this work will be a comparison of the two approaches on other types of damage. This will require carrying out new experiments, where other different damage mechanisms are introduced on the tie-rods, e.g., the failure of the constraints.

## Figures and Tables

**Table 1:** First case study: different stages of the corrosion process.

Label	Date	Δh [%]	Photo
A1	24 November 2020	6	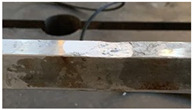
B1	8 February 2021	8	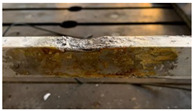
C1	8 April 2021	22	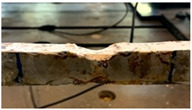
D1	25 May 2021	28	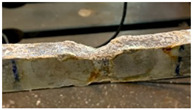

**Table 2:** Second case study: different stages of the corrosion process.

Label	Date	Δh [%]	Photo
A2	15 March 2021	2	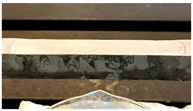
B2	9 April 2021	5	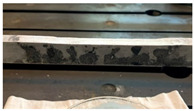
C2	29 April 2021	6	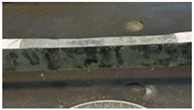
D2	25 May 2021	10	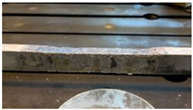

## Data Availability

The data presented in this study are available on request from the corresponding author.

## Appendix A

According to the SDOF approach, the modal analysis is aimed at extracting the properties of one of the vibration modes at a time [39]. The basic assumption is that, in the vicinity of a resonance, the behaviour of the system is dominated by the dynamics of the corresponding mode. All the modes in the frequency range of interest are sequentially analysed, rather than simultaneously. The principal limitation to such simple approach is that it applies only to structures that show lightly coupled modes, not closely spaced in frequency and not heavily damped.

The best fitting approach was adopted in this work, between the experimental power spectrum of the response measured by a single accelerometer and the analytical expression of the power spectrum of the response of an SDOF mechanical system with eigenfrequency $f_{m}$ excited by white noise. Considering a range of frequencies $\Delta_{m}$ around $f_{m}$ where the SDOF hypothesis is assumed ($\Delta_{m}$ is the width of the analysed range of frequencies), the procedure can be resumed in the following steps:

- The experimental power spectrum of the response ${\hat{G}}_{yy}\left(\omega \right)$, function of the angular frequency ω (ω=2πf, where *f* is the frequency expressed in Hertz) is calculated through Welch’s method, i.e., a frequency averaging approach. In this work, the identification was carried out every hour using sub-records duration equal to 40 s, overlap of 50% and a Hanning window.
- The power spectrum of the response of an SDOF model is considered, which is defined according to the following expression [39]:
  (A1) $$G_{yy}\left(\omega ,f_{m},\xi_{m},Q_{m},B_{m}\right)={\left|\frac{Q_{m}}{-\omega^{2}+j2\xi_{m}\left(2\pi f_{m}\right)\omega +{\left(2\pi f_{m}\right)}^{2}}+B_{m}\right|}^{2}$$
  where j is the imaginary unit, $\xi_{m}$ is the *m*-th modal damping ratio, $Q_{m}$ is a constant (function of the white noise level, the eigenvector component at the measurement point and the modal participation factor) and $B_{m}$ is the contribution of the out-of-band modes (for notation convenience, the parameters are grouped in the vector ${\underline{\alpha }}_{m}=\left[f_{m},\xi_{m},Q_{m},B_{m}\right]$, such that $G_{yy}\left(\omega ,f_{m},\xi_{m},Q_{m},B_{m}\right)=G_{yy}\left(\omega ,{\underline{\alpha }}_{m}\right)$).
- The simplex search method [61] is adopted to solve the minimization problem described by the following expression:
  (A2) $$\min_{{\underline{\alpha }}_{m}}=\sum_{h=h_{\min}}^{h_{\max}}{\left({\hat{G}}_{yy}\left(\omega_{h}\right)-G_{yy}\left(\omega_{h},{\underline{\alpha }}_{m}\right)\right)}^{2}$$
  where $h_{\min}$ and $h_{\max}$ are counters on frequency values which point at the lower and upper limit of a frequency range of amplitude equal to $\Delta_{m}$ around $f_{m}$, respectively.

For each of the considered eigenfrequencies, the initial values for $f_{m}$ and $\Delta_{m}$ can be defined through a visual inspection of the power spectrum. First attempt values for the minimization step can be obtained, e.g., by searching for the frequency at which the maximum of ${\hat{G}}_{yy}\left(\omega \right)$ is observed, for the eigenfrequency, and by adopting the peak-picking technique [39], for the modal damping ratio.

## Appendix B

The EM algorithm starts with an initial model characterized by parameters $\lambda^{\left(z\right)}$ and estimates a new model characterized by $\lambda^{\left(z+1\right)}$, such that $p\left(\left[x\right]|\lambda^{\left(z+1\right)}\right)>p\left(\left[x\right]|\lambda^{\left(z\right)}\right)$, iterating the procedure until a certain threshold on the model likelihood is reached. The EM algorithm consists of two steps (expectation and maximization) which guarantee a monotonic increase in the model likelihood [60,62].

In the expectation step, the model parameters $\lambda^{\left(z\right)}$ are considered as fixed and the posterior probability is calculated for every feature vector ${\underline{x}}_{r}$ and for every cluster *i*:

(A3) $$P_{ri}\left({\underline{x}}_{r}|{\underline{\mu }}_{i},{\left[\Sigma \right]}_{i}\right)=\frac{\theta_{i}N\left({\underline{x}}_{r}|{\underline{\mu }}_{i}^{\left(z\right)},{\left[\Sigma \right]}_{i}^{\left(z\right)}\right)}{\sum_{v=1}^{K}\theta_{v}N\left({\underline{x}}_{r}|{\underline{\mu }}_{v}^{\left(z\right)},{\left[\Sigma \right]}_{v}^{\left(z\right)}\right)}$$

which is the a posteriori probability that the feature vector ${\underline{x}}_{r}$ belongs to the cluster *i*. The posterior probability of all the samples in each Gaussian component is calculated according to the following expression:

(A4) $$P_{i}=\sum_{r=1}^{R}P_{ri}\left({\underline{x}}_{r}|{\underline{\mu }}_{i},{\left[\Sigma \right]}_{i}\right)$$

In the maximization step, the model parameters are updated, obtaining $\lambda^{\left(z+1\right)}$ according to the following expressions:

(A5) $$\theta_{i}^{\left(z+1\right)}=\frac{P_{i}}{R}$$

(A6) $${\underline{\mu }}_{i}^{\left(z+1\right)}=\frac{\sum_{r=1}^{R}\left(P_{ri}\left({\underline{x}}_{r}|{\underline{\mu }}_{i},{\left[\Sigma \right]}_{i}\right){\underline{x}}_{r}\right)}{P_{i}}$$

(A7) $${\left[\Sigma \right]}_{i}^{\left(z+1\right)}=\frac{\sum_{r=1}^{R}\left(P_{ri}\left({\underline{x}}_{r}|{\underline{\mu }}_{i},{\left[\Sigma \right]}_{i}\right){\left({\underline{x}}_{r}-{\underline{\mu }}_{i}^{\left(z+1\right)}\right)}^{T}\left({\underline{x}}_{r}-{\underline{\mu }}_{i}^{\left(z+1\right)}\right)\right)}{P_{i}}$$

A commonly adopted stopping criterion is defined on the log-likelihood increment between two subsequent iterations, according to the next expression:

(A8) $$\left|\frac{\log\left(L\left(\lambda^{\left(z+1\right)}\right)\right)}{\log\left(L\left(\lambda^{\left(z\right)}\right)\right)}\right|-1<\epsilon$$

where ϵ is a user defined tolerance (a tolerance of 1e-6 is adopted in this work); otherwise the steps are recursively performed until the maximum number of iterations is reached (a maximum number of iterations equal to 500 is used in this work).

The EM algorithm is strongly dependent on the initialization, i.e., on the choice of model parameters λ for the first iteration. Common solutions are the use of multiple random starting points and then choosing the final estimate with the highest likelihood, or initializing by other clustering algorithms, e.g., the K-means algorithm [63] (the latter approach is that adopted in this work). Due to the high sensitivity to the initialization, the convergence of the EM algorithm is guaranteed to a local maximum but not to a global maximum [60].
